# tRNA anticodon shifts in eukaryotic genomes

**DOI:** 10.1261/rna.041681.113

**Published:** 2014-03

**Authors:** Hubert H. Rogers, Sam Griffiths-Jones

**Affiliations:** Department of Life Sciences, The University of Manchester, Manchester M13 9PT, United Kingdom

**Keywords:** transfer RNA anticodon, covariation, genetic code

## Abstract

Embedded in the sequence of each transfer RNA are elements that promote specific interactions with its cognate aminoacyl tRNA-synthetase. Although many such “identity elements” are known, their detection is difficult since they rely on unique structural signatures and the combinatorial action of multiple elements spread throughout the tRNA molecule. Since the anticodon is often a major identity determinant itself, it is possible to switch between certain tRNA functional types by means of anticodon substitutions. This has been shown to have occurred during the evolution of some genomes; however, the scale and relevance of “anticodon shifts” to the evolution of the tRNA multigene family is unclear. Using a synteny-conservation–based method, we detected tRNA anticodon shifts in groups of closely related species: five primates, 12 *Drosophila*, six nematodes, 11 Saccharomycetes, and 61 Enterobacteriaceae. We found a total of 75 anticodon shifts: 31 involving switches of identity (alloacceptor shifts) and 44 between isoacceptors that code for the same amino acid (isoacceptor shifts). The relative numbers of shifts in each taxa suggest that tRNA gene redundancy is likely the driving factor, with greater constraint on changes of identity. Sites that frequently covary with alloacceptor shifts are located at the extreme ends of the molecule, in common with most known identity determinants. Isoacceptor shifts are associated with changes in the midsections of the tRNA sequence. However, the mutation patterns of anticodon shifts involving the same identities are often dissimilar, suggesting that alternate sets of mutation may achieve the same functional compensation.

## Abstract (precis)

Embedded in the sequence of each transfer RNA are elements that promote specific interactions with its cognate aminoacyl tRNA-synthetase. Because the anticodon is often a major identity determinant itself, it is possible to switch between certain tRNA functional types by means of anticodon substitutions. By using a synteny-conservation–based method, we detected tRNA anticodon shifts in groups of closely related species and found a total of 75 anticodon shifts: 31 involving switches of identity (alloacceptor shifts) and 44 between isoacceptors that code for the same amino acid (isoacceptor shifts).

## INTRODUCTION

Transfer RNAs (tRNA) are involved in two functionally related but physically decoupled reactions that are integral to protein synthesis (Söll and RajBhandary 1995). First, the amino acid identity of a tRNA molecule is determined by its recognition and aminoacylation by an aminoacyl tRNA-synthetase (aaRS) enzyme (Goodman and Rich 1962). Specific recognition by a synthetase is facilitated by the tRNA's sequence and structural features (McClain 1993; Giege et al. 1998). The second reaction entails donation of the charged amino acid at the ribosome, following mRNA codon recognition by the tRNA anticodon (Ogle et al. 2001). Together these two reactions determine the sequence of protein. Translational accuracy is therefore dependent on the correspondence of a tRNA's identity and its anticodon and on their accurate reflection of the genetic code.

tRNAs are ∼76 nt in length (Marck and Grosjean 2002). The number of residues in stem and loop regions is sufficiently conserved so that they can be referenced by a standard numbering system (Sprinzl et al. 1991). Length differences occur in the variable loop and the D-loop. In many eubacteria and archaea and in virtually all eukaryotes, residues 74–76 (nearly always CCA) are added post-transcriptionally and are not coded for by the tRNA genes (Deutscher and Ni 1982). The amino acid is attached to base A76 in the acylation reaction (Delagoutte et al. 2000; Xiong and Steitz 2004). Residue 73 at the end of the acceptor stem is unpaired in all tRNAs except tRNA-His, which in eukaryotes contains a G residue post-transcriptionally added to the 5′ terminus (position −1). The anticodon residues are positions 34, 35, and 36.

While all aaRSs catalyze the same type of reaction by the same mechanism, they share only a few common structural features. The synthetase family of enzymes can be divided into two distinct classes on the basis of the architecture of the catalytic domain (Eriani et al. 1990). Class I synthetases approach the tRNA acceptor stem from the minor groove and aminoacylate the 2′ hydroxyl of the terminal A nucleotide, whereas class II synthetases approach from the major groove side and generally aminoacylate the 3′ hydroxyl (Lapointe and Brakier-Gingras 2003). The aminoacylation reaction involves two transition states that are stabilized by the binding energy provided by aaRS–tRNA interactions (Loftfield and Vanderjagt 1972).

The 20 aaRS enzymes must discriminate between a pool of structurally similar tRNAs. Therefore specific elements in tRNA sequence and structure must be present to allow synthetases to recognize and aminoacylate cognate tRNAs and avoid interaction with noncognate tRNAs. However, not all recognition elements (or “identity” elements) are obvious; many tRNAs that accept the same amino acid (isoacceptors) have different nucleotide sequences (Fender et al. 2004; Sprinzl and Vassilenko 2005), and even tRNAs with the same anticodon can have different recognition elements (Geslain and Pan 2010). To date, no complete set of identity elements is known for the entire tRNA complement of any organism.

The sequence and structural features that promote recognition by specific aaRSs are termed identity determinants, while those that prevent indiscriminate interactions are termed antideterminants (Giege et al. 1998). Known tRNA identity elements are mostly located at the two opposite ends of the tRNA molecule: the anticodon itself, the discriminator base (unhybridized position 73), and the terminal 4 bp of the acceptor stem. Position 37 in the anticodon loop is also involved in identities of tRNAs charged by class I synthetases (Giege et al. 1998). Identity elements in the tRNA core (nt 8–31 and 39–65) are more species-dependent and aaRS-tRNA system–dependent, are dispersed over numerous positions, and are individually less strong (Giege et al. 1998).

Identity determinants have been investigated using a variety of techniques for many years (Francklyn and Schimmel 1989; McClain 1993; Giege et al. 1998). Typically these analyses focus on one tRNA functional class for one model organism. Freyhult et al. (2007) point out that this approach can potentially reduce perspective on two important aspects of the identity problem: how the tRNA identity elements of different tRNA functional classes work together in the cell and how tRNA identity elements evolve among lineages for specific functional classes, and coevolve within lineages for different functional classes. It is known that some tRNAs have different identity rules in different taxa. For example *Escherichia coli* tRNA-Gly uses a crucial U73 discriminator base in combination with a C2:G71 pair, while in mammals the discriminator base is A73 (Shiba et al. 1994; Hipps et al. 1995); prokaryotic tRNA-Asp uses a U73 and the first base pair G1:C72 of the stem, while eukaryotes use A73 and the third base pair of the stem (Nameki et al. 1992, 1995). Although crucial to our understanding of the evolution of the genetic code (Schimmel et al. 1993), the evolution of tRNA identity elements has been the subject of few studies. The availability of numerous genome sequences and tRNA annotations allows the analysis of the tRNA identity system as a whole, and comparative genomics methods provide insight into tDNA sequence evolution.

The tRNA multigene family is partitioned functionally according to amino acid specificity such that each tRNA falls into one of the 22 standard proteinogenic amino acid accepting groups. Most functional groups comprise multiple isoacceptor tRNAs. A reasonable model of evolution for this scenario would be that each group arose from a single common ancestor with the corresponding amino acid identity. However, this classical model of evolution appears to be true for only some isoaccepting groups (Saks et al. 1998). The evolution of other tRNA groups requires more complex explanation. Phylogenetic analyses of *Escherichia coli* tRNAs have revealed that while some isoaccepting groups form discrete clusters, the isoacceptors of most groups are dispersed throughout the dendogram (Saks et al. 1998). It has therefore been suggested that some isoacceptors may be derived from different ancestors (Cedergren et al. 1981; Fitch and Upper 1987; Saks et al. 1998).

Several experiments have demonstrated a simple and plausible mechanism for a “gene recruitment” process, whereby a tRNA sequence is recruited to a different amino acid identity. It is possible in vitro to alter a tRNA's amino acid charging identity by anticodon mutations (Schulman and Pelka 1989; Pallanck and Schulman 1991). It has also been shown that mutations outside the anticodon, for example, involving acceptor stem nucleotides that are strong identity determinants, can also effectively switch the identity of a tRNA (Hipps et al. 1995). That “identity switches” could potentially occur by single or a few mutations was first demonstrated in vivo by the rescue of an inviable tRNA-Thr(UGU) deletion mutant of *E. coli* by a mutant tRNA-Arg(UGU) gene (Saks et al. 1998). A handful of studies have since inferred putative instances of tRNA identity switching events during the evolutionary history of organisms and organelles, in mitochondrial genomes (Rawlings et al. 2003; Lavrov and Lang 2005; Wu et al. 2012), in vertebrates (Coughlin et al. 2009; Tang et al. 2009), in *Drosophila* (Rogers et al. 2010), in nematodes (Hamashima et al. 2012), and in primates (Wang and Lavrov 2011). One recent study has implicated a tRNA-Arg(GCU) to ACU (normally the anticodon of tRNA-Trp) mutation in a patient with mitochondrial encephalomyopathy (Roos et al. 2013).

By using a micro-synteny–based method based on our previous work to identify orthologs of the tRNAs in the *Drosophila* genus, we have carried out an extensive search for instances of tRNA gene recruitment events. We searched for sets of tRNA orthologs in complete genome sequences of five primates, six nematode worms, 12 *Drosophila* flies, 11 Saccharomycetes yeast, and 61 Enterobacteriaceae bacteria. We identified instances of tRNA orthologs with different anticodons (anticodon shifts), gene recruitment events involving anticodons of both different tRNA identities (alloacceptor shifts), and anticodon mutations that remain associated with the same amino acid (isoacceptor shifts). In order to further study the structural mechanisms of both types of tRNA anticodon shift, we searched for mutations in other regions of the tRNA that covary with the mutations in the anticodon.

## RESULTS

### Mappings

We predicted tRNA genes in the genomes of five groups of species: five primates, 12 flies (*Drosophila*), six nematode worms (five *Caenorhabditis* and one *Pristionchus*), 11 Saccharomycetes yeast (10 *Saccharomyces* and one *Candida*), and 61 bacteria from the family Enterobacteriaceae, each one from a different genus (for list of species, see Supplemental File 1). Among the five groups of organisms, there is great variety in the size of the genomes, the number of predicted tRNAs, and the evolutionary divergences involved (Table 1; Supplemental File 2).

**TABLE 1.**
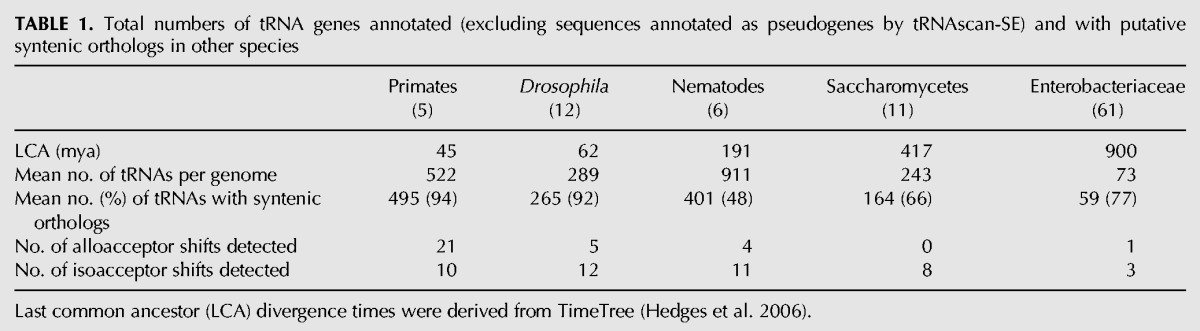
Total numbers of tRNA genes annotated (excluding sequences annotated as pseudogenes by tRNAscan-SE) and with putative syntenic orthologs in other species

We used sequence similarity in genomic regions flanking the tRNAs to identify potential tRNA orthologs in different species. A tRNA may have multiple mappings to another genome; this may mean that multiple orthologs of that gene in another species have arisen by duplication or could be due to spurious similarity matched in tRNA flanking regions caused by undetected and unmasked repeat sequences. For each taxonomic group, we compiled a list of ortholog sets by applying single-linkage clustering to the tables of mappings. Table 1 shows that the number of tRNAs with syntenic orthologs in other species vary considerably: >90% for the primates and the *Drosophilids*, 68% for the Saccharomycetes tRNAs, and only 44% for the nematode worm tRNAs. In general, high numbers of tRNAs mapped between genomes indicate that the tRNA complements are highly conserved between species. The low synteny conservation among the nematode tRNAs suggests either a high rate of tRNA gene turnover or a recent trend of tRNA gene proliferation. On the other hand, the yeast and bacterial tRNA complements could be considered as relatively stable, especially given the greater scales of evolutionary divergence within those groups.

### Anticodon shifts

We used our sets of predicted tRNA orthologs to identify potential changes in anticodon sequences. The criteria employed here to identify so-called anticodon shifts are similar to those reported previously (Rogers et al. 2010). However, in order to study the structural changes associated with tRNA anticodon shifts, we have chosen to restrict ourselves to a high-confidence set of potential shifts. To that end, we have focused on shifts that have occurred since the last common ancestor of the clade and that have supporting evidence from more than one test (see Materials and Methods). These criteria are therefore stricter than those previously used by us and others (Rogers et al. 2010; Wang and Lavrov 2011).

A total of 233 ortholog sets were found to contain tRNAs with different anticodon sequences (for details of the anticodon shift-containing ortholog sets for each taxa, see Supplemental File 3). After removing low-confidence orthologs with dissimilar sequences and removing tRNAs with unstable structures (see Materials and Methods), we obtained a filtered set of 75 predicted anticodon shifts: 31 alloacceptor shifts (involving a predicted change in tRNA identity) and 44 isoacceptor shifts (a change between anticodons specifying the same identity).

We considered a typical eukaryotic tRNA complement of 46 tRNA anticodons representing 21 identities (the 20 directly encoded identities and selenocysteine) (Marck and Grosjean 2002). Within this set, there are 414 possible single-base anticodon substitutions: 299 of these would produce an anticodon coupled to a codon specifying a different identity, 101 the same identity, and 14 changes to anticodons complementary to STOP signals (suppressor mutations). Thus if the anticodon shifts occurred by chance with no constraint, we would expect approximately three times as many alloacceptor shifts as isoacceptor shifts. However, we observe slightly more isoacceptor shifts, suggesting significantly greater constraint on alloacceptor shifts (*P* < 0.0001, χ^2^ test).

In total, there are 71 anticodon shifts involving single-base anticodon changes (43 isoacceptor shifts and 28 alloacceptor shifts) (Table 2), and four two-base changes (three of which are alloacceptor shifts). Substitutions of the middle anticodon base (position 35) are most common in alloacceptor shifts, followed by the third anticodon position. Thirty-nine out of the 44 isoacceptor shifts are substitutions of the first anticodon base. The single isoacceptor shift involving two anticodon base changes involves a switch between tRNA-Leu(AAG) and tRNA-Leu(UAA) in nematodes. Visual inspection of the alignment suggests an insertion or deletion of one base in the anticodon loop is responsible, rather than two independent substitutions. The most frequent substitution is between U and C (24 of 79) (Table 3), which likely reflects the unique ability of first position anticodon U bases to pair with both A and G bases in the third codon position. Twenty-six percent of alloacceptor shifts and 39% of isoacceptor shifts involve switches between these bases. The identity of the bases involved in alloacceptor shift mutations appears to be much more evenly distributed than isoacceptor changes (Table 3). None of the shifts we detect involve initiator tRNAs.

**TABLE 2.**
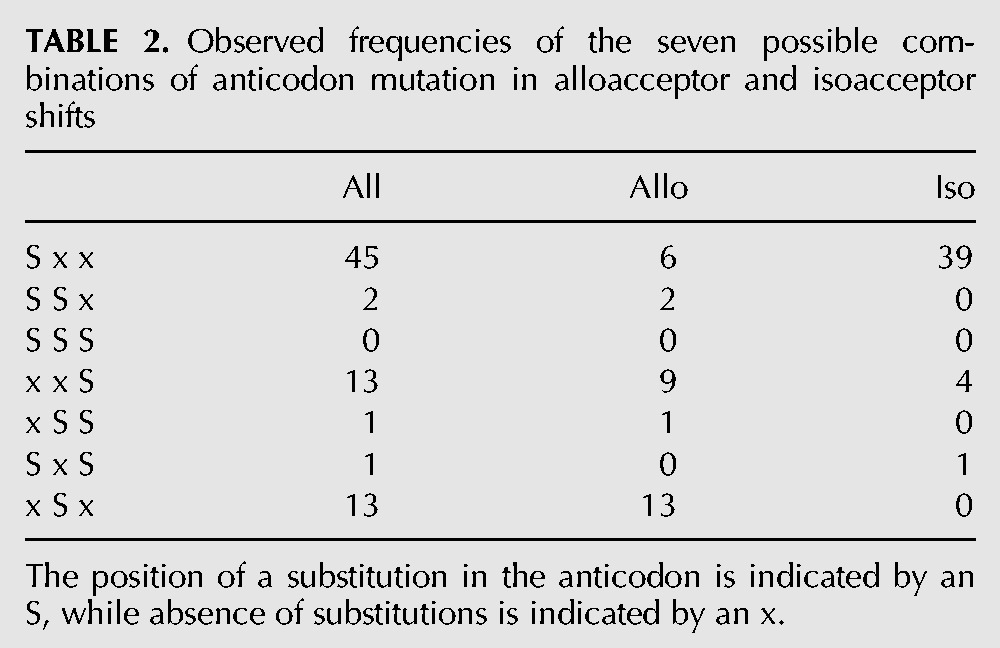
Observed frequencies of the seven possible combinations of anticodon mutation in alloacceptor and isoacceptor shifts

**TABLE 3.**
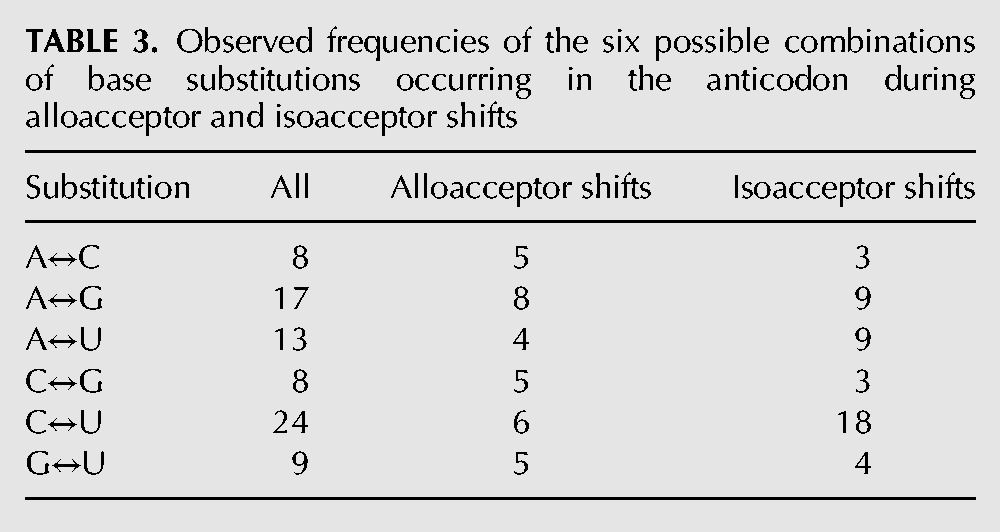
Observed frequencies of the six possible combinations of base substitutions occurring in the anticodon during alloacceptor and isoacceptor shifts

Table 1 shows the numbers of alloacceptor and isoacceptor anticodon shifts detected in each taxonomic group. The numbers of anticodon shifts correlates with the average number of mapped tRNA genes present in that group (Pearson's *r* = 0.88) and with the average genome size (Pearson's *r* = 0.89). Putative primate anticodon shifts were the most common, comprising 41% of the total detected, despite this group containing only five genomes and the shortest evolutionary divergence of the groups tested. In contrast, no shifts were detected in yeast, and only one shift was detected in Enterobacteriaceae, despite the numerous species in the latter data set (61) and their much higher evolutionary divergences. Despite the high divergence time, lack of orthologous tRNA identifications is not responsible: Our synteny strategy identifies orthologs for 77% of tRNAs in these bacteria. Thus we suggest that the relative absence of anticodon shifts is related to the lower redundancy of tRNAs in these genomes. In *Drosophila*, nematode worms, Saccharomycetes, and Enterobacteriaceae, we found a greater number of isoacceptor shifts than alloacceptor shifts, at least twice as many in each taxonomic group. However, in primates this trend is reversed; we identified twice as many potential alloacceptor shifts as isoacceptor shifts. The reason behind this excess of alloacceptor shifts in primates is obscure.

Among the 30 eukaryotic alloacceptor anticodon shifts, 17 involve a transition between anticodons that couple with different classes of aaRS classes, eight shifts switch between different class I synthetases, and five between class II synthetases (Table 4). The high number of synthetase “transition” shifts is not attributable to clustering of the anticodons that each synthetase class uses; by random single-base substitutions, class I and class II synthetase-coupled anticodons have similar likelihood of switching to an anticodon associated with the same or different synthetase classes. (Averaged over all identities, the numbers of distinct alloacceptor shifts possible by random single base substitution for the tRNAs of a given class are as follows: class I→class I, 3.5; class I→class II, 3.8; class II→class I, 4.2; and class II→class II, 3.8.) The observed set of alloacceptor shifts include 27 (33%) of the 81 different identity pairs that are possible to achieve with single anticodon base mutations. For 12 synthetase transition identity shifts, we can predict the direction of the shift by parsimony: Eight are from class II to class I synthetase identities, and four are from class I to class II synthetase identities.

**TABLE 4.**
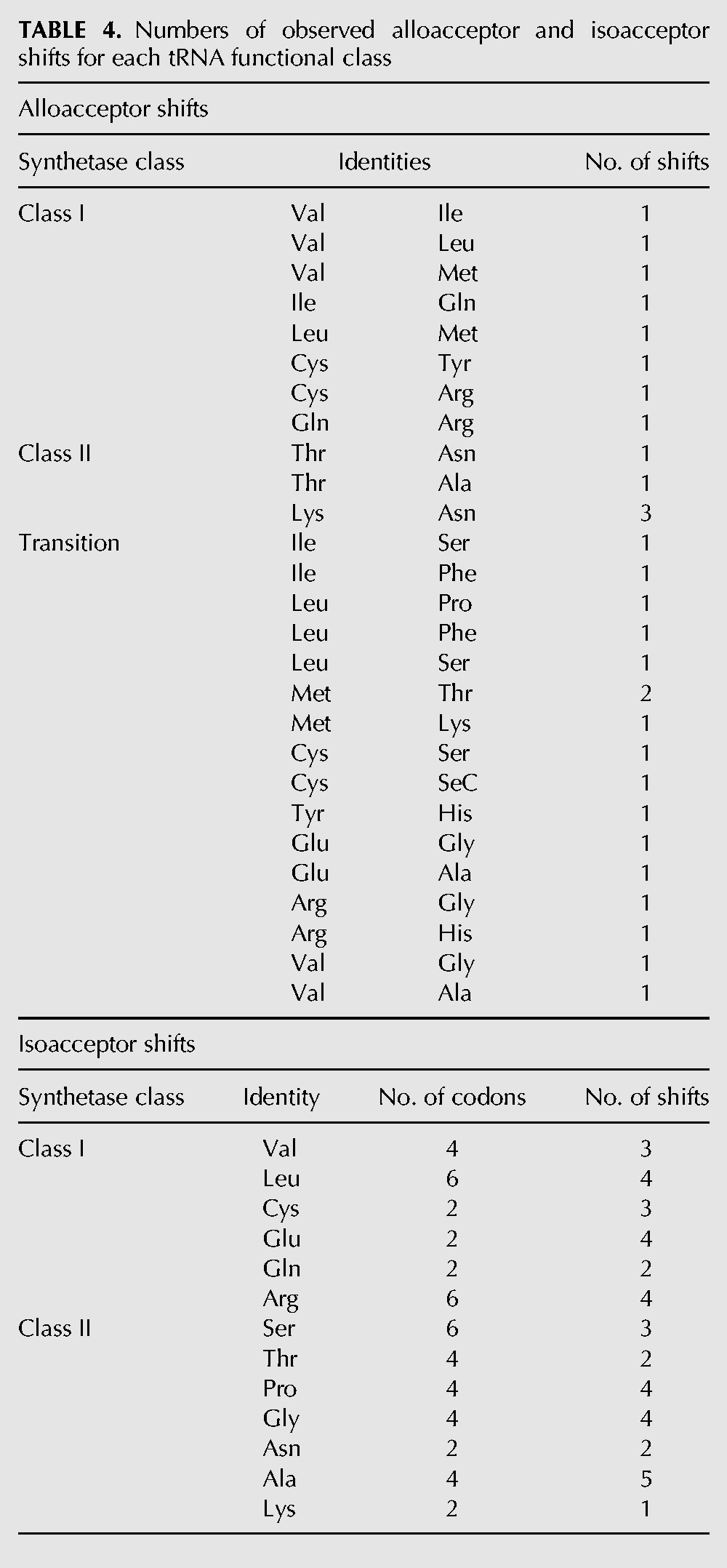
Numbers of observed alloacceptor and isoacceptor shifts for each tRNA functional class

We found isoacceptor shifts involving 14 out of 19 capable tRNA identities (all except Asp, His, Ile, Met, and Tyr; note that Trp and SeC identities have only one tRNA alloacceptor). In contrast to the alloacceptor shifts, the isoacceptor shifts show no bias in the synthetase classes in which they occur. Among the 44 isoacceptor switches, we observe 22 in class I synthetase identities and 22 in class II synthetase identities (Table 4). There is a positive correlation between the number of anticodons in an isoacceptor family and the number of shifts observed between those anticodons (*P* = 0.04, Pearson's correlation).

### Covarying mutations

Among orthologous tRNA families, we find large variation in the number of extra-anticodon mutations and in the positions of these mutations. By using pair-entropy normalized mutual information (MI) as a measure of covariation with the anticodon, we quantified the covariance occurring at each site with respect to the anticodon, across all alignments that have anticodon shifts (see Materials and Methods; for details of calculated covariance in each anticodon shift alignment, see Supplemental File 4). The summed MI score across all alignment positions for an anticodon shift is a measure of the number of mutations (including insertions and deletions) at bases other than the anticodon that covary with the anticodon mutation(s). Since prokaryotic aaRS-tRNA systems differ considerably from eukaryotic systems and since we have detected few anticodon shifts in the bacterial data set, the following analyses focus on the eukaryotic data sets only.

#### Alloacceptor shifts

The 30 eukaryotic alloacceptor anticodon switches have a mean summed MI score of 3.9. This varies greatly among the synthetase classes involved (*P* = 0.018, ANOVA): Class I synthetase identity switches have an average MI score of 6.3, and switches among class II identities only 2.2. Switches between the two synthetase classes (trans identity shifts) have an average MI of 3.2. The large majority of eukaryotic alloacceptor shifts (25 out of 30) involve a unique pair of identities. The alloacceptor shift identity pairs that occur more than once comprise two Met/Thr shifts and three Lys/Asn shifts.

**FIGURE 1.**
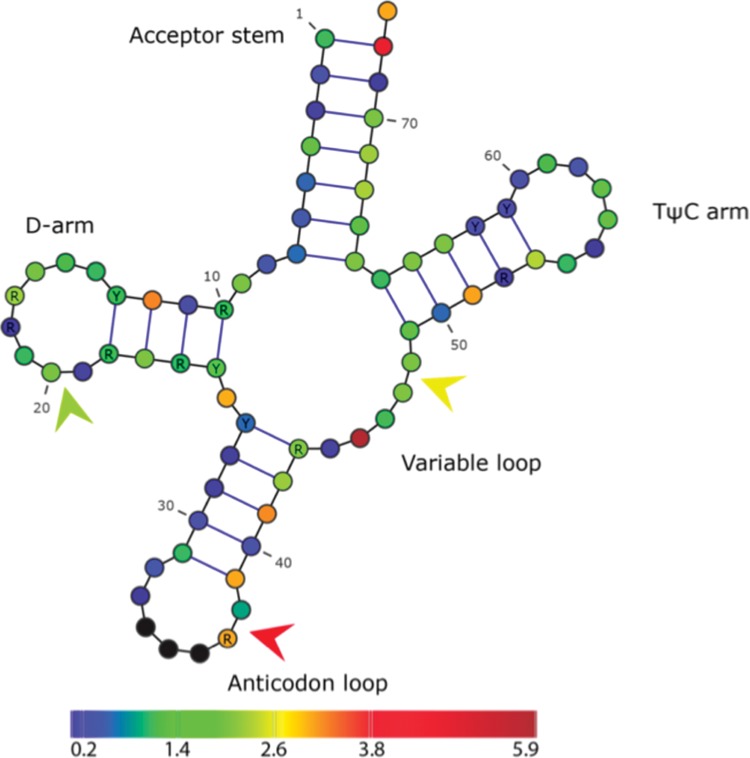
Alloacceptor shift covariance heat map showing how extra-anticodon mutations covary with anticodon changes. Color indicates the SD from the alloacceptor consensus mean of summed MI (∑MI) at each position: dark blue, <−1 SD; blue, −1 SD; green, mean; yellow, +1 SD; bright red, +2 SD; and dark red, >+2 SD. The nonconsensus positions 20/21, 27/28, and 47/48 are indicated by arrows.

We summed the MI score contributions of all alloacceptor shifts at each structural position, each alignment position thus having a total MI score (**∑**MI) that reflects the strength of its covariance with the anticodon over all alloacceptor shifts. The data show that there are substantial differences in covariance at different positions in the consensus tRNA model (Fig. 1). Nine consensus positions and the frequently covarying nonconsensus position 37/38 have a summed MI score >1 standard deviation (SD = 1.19) from the mean (1.36) (Fig. 1). The nonconsensus position 37/38 is the site of introns in some eukaryotic tRNAs (Marck and Grosjean 2002), and we see both insertions and substitutions at these positions.

The positions that covary most with anticodon mutations are located across all domains of the tRNA molecule. Five of the 10 top-scoring sites (37, 27/38, 39, 72, and 73) are located in the anticodon and acceptor domains; these two distal ends of the structure are directly involved in the key interface functions of the tRNA molecule and are known to be important identity determinants for several aaRS-tRNA systems (Giege et al. 1998). However, the remaining five positions are not known as primary identity elements. In particular, the T, D, and variable domains have important structural roles (Marck and Grosjean 2002) but are typically known to harbor minor or partial identity elements that are used in only one tRNA system (Giege et al. 1998). These results therefore suggest more complex structural rearrangements that involve coordinated mutation of other domains of the tRNA molecule are required for different aaRS systems or for interaction with the ribosome, for example (Buchan et al. 2006; Saks and Conery 2007).

Perhaps the most striking feature of the alloacceptor shift mutations is their heterogeneity. None of the top-scoring positions appears heavily biased toward any particular pair of identities, or any group of species (for details of mutations at these sites, see Table 5). Only one site appears biased toward a class of synthetase (position 12, class I), although the number of shifts (four) is low. The highest-scoring position (45) covaries with the anticodon in seven different alloacceptor shifts: only 23% of the 30 detected in eukaryotes.

**TABLE 5.**
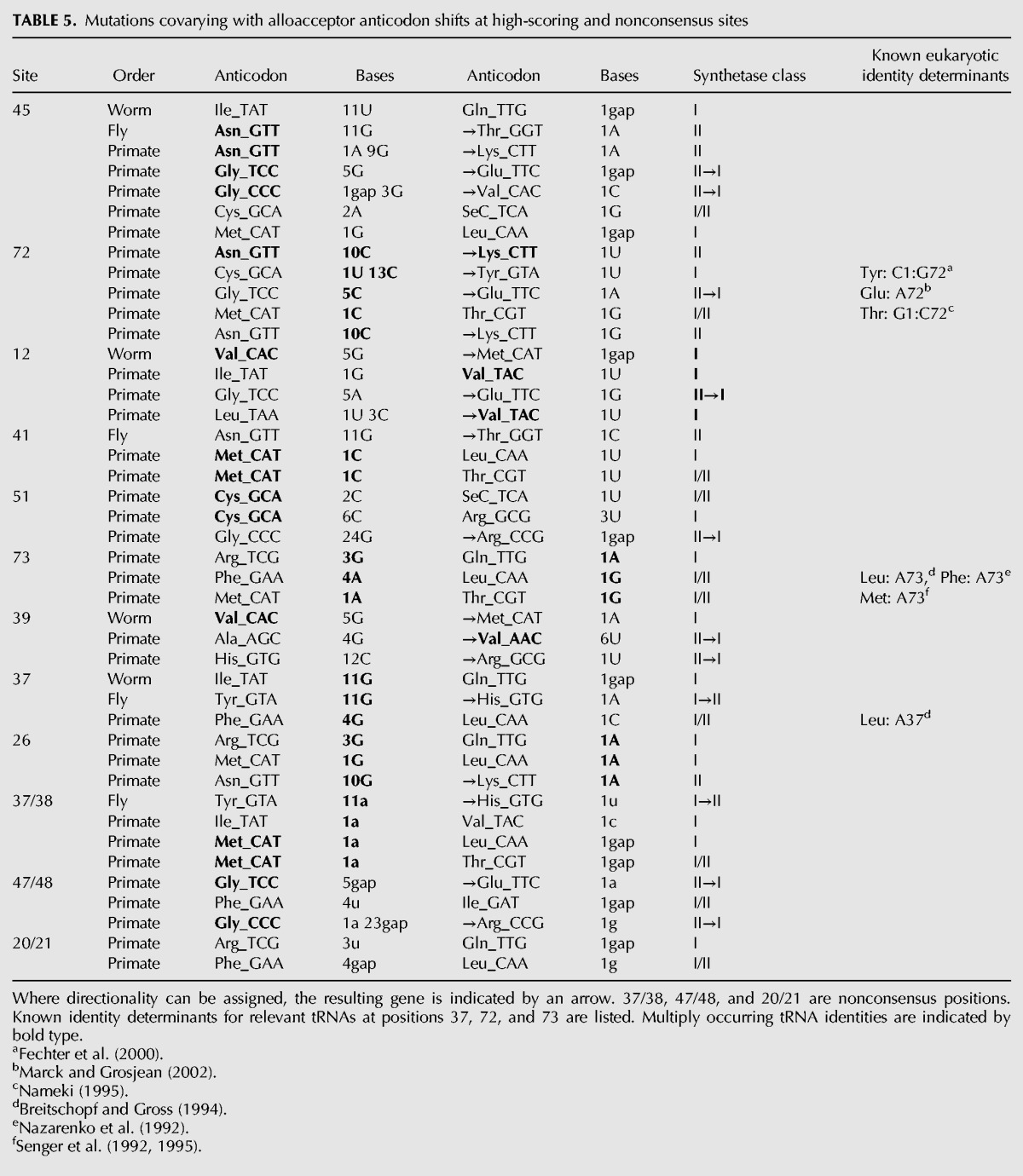
Mutations covarying with alloacceptor anticodon shifts at high-scoring and nonconsensus sites

Out of the 30 alloacceptor shifts in eukaryotes, only two pairs of identities appear more than once: Asn/Lys three times and Met/Thr twice. One of the three Asn/Lys identity shifts, occurring in *Drosophila*, consists of only a GTT > TTT anticodon substitution with no extra-anticodon mutations. tRNA-Lys identity is known to be largely dependent on the anticodon in mammalian aaRS-tRNA systems (Stello et al. 1999; Francin and Mirande 2006), so no further mutations may be required for lysine charging of this tRNA. The remaining two Asn/Lys shifts are GTT-to-CTT anticodon mutations, both in primates, but are associated with different extra-anticodon mutations. The only mutation site common to both, position 72, involves substitutions of C for different bases: U and G. Similarly the two Met/Thr shifts, both involving Met(CAT) and Thr(CAA), have different associated mutations. One occurs in fly and has no strongly covarying mutations outside the anticodon substitution, and the other is in primates, with five extra-anticodon mutations.

For the alloacceptor shifts with covarying mutations visible at major identity determining sites 37, 72, and 73, we compared these mutations with known eukaryotic identity determinants reported in the literature (Table 5). Out of nine unique shifts, three appear to coevolve according to known identity rules. One shift matches a likely identity determinant for tRNA-Glu (A72) (Marck and Grosjean 2002). Two shifts involving tRNA-Lys and tRNA-Asn do not contradict any known identity determinants, since the posterior state tRNA-Lys identity is dependent mainly on the anticodon (Stello et al. 1999; Francin and Mirande 2006). Out of the remaining six shifts, two have mutations that contradict the known identity determinants of one anticodon state: These are for tRNA-Tyr (G72) (Lee and RajBhandary 1991) and tRNA-Leu (A73 and A37) (Breitschopf and Gross 1994; Breitschopf et al. 1995). For the remaining four, we found no relevant identity determinant data to support or contradict coevolution at these sites.

#### Isoacceptor shifts

Of the 41 isoacceptor shifts identified in eukaryotes, we note that Glu, Gln, Leu, Lys, Ser, Gly, and Pro each have at least one isoacceptor shift without any extra-anticodon mutations that strongly covary with the anticodon. However, on average, the isoacceptor shifts have approximately the same number of extra-anticodon mutations as the alloacceptor shifts (average total MI, 4.1 and 3.9, respectively). This is perhaps a surprise and indicates the presence of anticodon-dependent structural constraints in the tRNA molecule separate from those imposed by the aaRSs.

**FIGURE 2.**
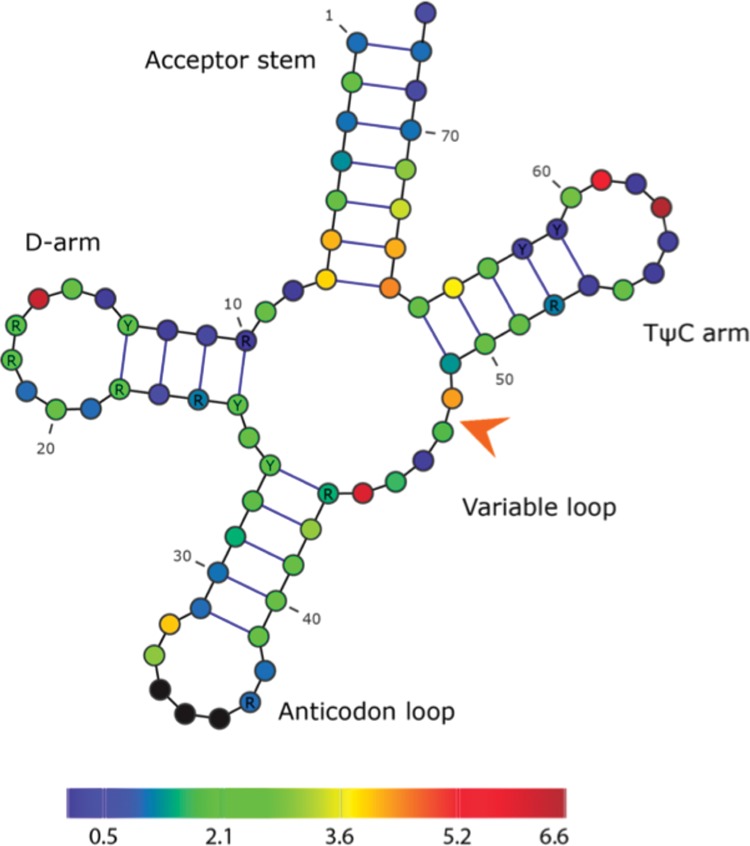
Isoacceptor shift covariance heat map showing how extra-anticodon mutations covary with anticodon changes. Color indicates the SD from the alloacceptor consensus mean of summed MI (∑MI) at each position: dark blue, <−1 SD; blue, −1 SD; green, mean; yellow, +1 SD; bright red, +2 SD; and dark red, >+2 SD. The nonconsensus position 47/48 is indicated by an arrow.

There are 11 consensus positions that have a total MI score of >1 SD (1.57) above the mean (2.06), together with a single nonconsensus position between positions 47 and 48, which is associated with multiple isoacceptor shifts (Fig. 2). The location of the sites that covary most with the anticodon is very different for isoacceptor shifts top-scoring compared with alloacceptor shifts (Fig. 2). For isoacceptor shifts, the distal end of the acceptor stem and the anticodon loop generally have low MI scores; instead, the top-scoring sites are almost all located in the middle sections of the tRNA structure, which in general are not associated with previously described identity elements. The four highest-scoring sites are located in loop regions: of the D (16), T (57 and 59), and variable loops (44). Four further high-scoring sites (six, seven, 66, and 67) are located at the base of the acceptor stem. Only one high-scoring site is located at distal end of the molecule: position 32 on the 5′ side of the anticodon loop, which is noteworthy since in alloacceptor shifts covarying mutations in the anticodon loop and stem tend to be on the opposite 3′ side.

The variable loop has two covarying consensus positions, 44 and 48. Interestingly, in alloacceptor shifts the adjacent position 45 is the predominant site of covarying mutations, while position 44 has none. The only nonconsensus position that is a common mutation site in the isoacceptor shifts is also located in the variable loop. Position 47/48 is mutated in five isoacceptor shifts, only one involving an insertion or deletion and the remaining four being substitutions (Fig. 2). The variable domain is thus the only structural region that is strongly associated with both alloacceptor and isoacceptor shifts.

Like the alloacceptor shifts, the most frequently mutated positions among isoacceptor shifts are all heterogeneous in tRNA identity (Table 6). They also comprise different synthetase classes, except position 6, which covaries with the anticodon only in class I synthetase tRNAs. There are a few visible trends in the covarying mutations when we consider which bases are being mutated. For example, although the identities involved are different, five different sites have covarying mutations that are consistent in the bases that are mutated: seven isoacceptor shifts contributing to the top-scoring site 57 undergo A/G substitutions; five out of six mutations at position 66 (hybridized site) are U/C substitutions; all five substitutions at position 7 (hybridized site) are between A and G bases; and position 48 mutations all involve C, while at the adjacent nonconsensus position 48/47 all involve U residues. These examples suggest that evolution at these sites is constrained in a consistent manner among the different tRNA identities.

**TABLE 6.**
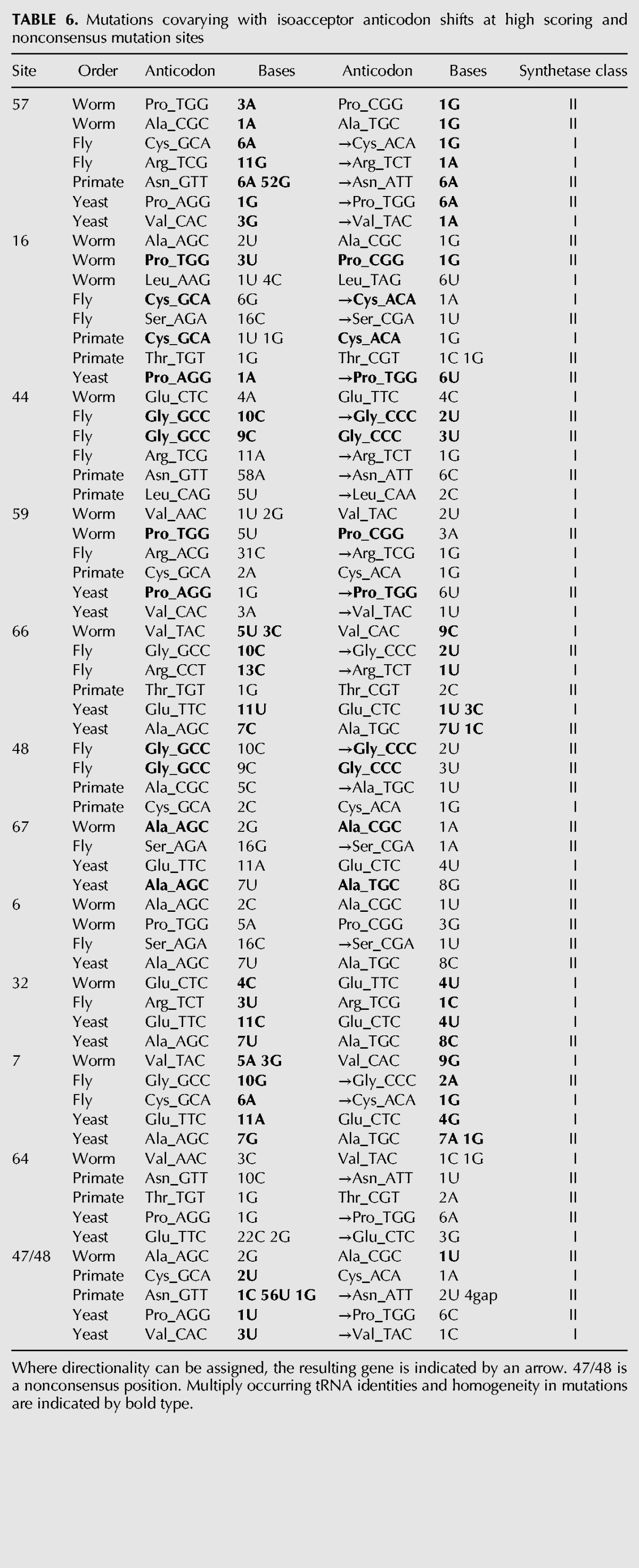
Mutations covarying with isoacceptor anticodon shifts at high scoring and nonconsensus mutation sites

## DISCUSSION

The tRNA multigene family comprises very short genes, which can be very highly conserved and yet are subject to high turnover (Bermudez-Santana et al. 2010; Rogers et al. 2010). Using sequence similarity methods, it is hard to justify a similarity threshold that both includes diverging orthologs and discards nonorthologous sequences, which may only differ by a few nucleotides. A purely phylogenetic approach is unlikely to reliably identify the close orthologs of a tRNA gene that has undergone an anticodon shift, since it may have accumulated several additional mutations, some of which may be convergent to the sequence patterns of another functional class. We used a synteny-mapping approach to finding tRNA sets of orthologs for the purpose of describing putative anticodon shifts.

Our analysis relies on the accurate prediction of tRNA genes and their identities. We use the tool tRNAscan-SE (Lowe and Eddy 1997), which has been shown to be highly sensitive and specific. However, we cannot rule out the possibility that some predicted tRNA genes are pseudogenes, are not actively transcribed, and thus are free to accumulate mutations. However, our method requires that tRNA genes have extant orthologs and therefore only includes tRNAs that are syntenically conserved in at least one other genome. While we expect that most conserved tRNA genes are likely to be transcribed and functional, we further attempted to minimize the effect on our analyses of such potential “inactive” tRNA genes by restricting inclusion in the ortholog sets to genes that were annotated with high scores, indicating a high degree of conformity with the consensus structure.

We have detected tRNA anticodon shifts in all taxonomic groups tested: primates, flies, worms, yeast, and bacteria. We find the largest number of anticodon shifts between five primate species (31; 41% of the total set of shifts identified), while only four were detected in the large group of 61 Enterobacteriaceae. The number of shifts detected in each group is correlated with the average size of that group's mapped tRNA gene complement. This suggests that anticodon mutations are facilitated by a high redundancy of tRNA gene in a genome. In species with high tRNA gene copy numbers, loss of one tRNA gene may have relatively little impact on overall tRNA population in the cell for that tRNA functional type.

### Coevolution in alloacceptor and isoacceptor anticodon shifts

In contrast with the random expectation, we detect more isoacceptor shifts than alloacceptor shifts in all species groups. This implies greater constraint on alloacceptor shifts than isoacceptor shifts, as might be expected. However, we find little difference between the categories in their average numbers of covarying mutations outside the anticodon, as quantified by MI (average ΣMI: 3.9 and 4.1, respectively). Mutations that coevolve with alloacceptor anticodon shifts are expected to ensure that the amino acid identity matches the new anticodon. Indeed, many observed mutations do match those sites commonly associated with tRNA identity determinants. However, extra-anticodon mutations associated with isoacceptor shifts are harder to rationalize and have not been previously described. We suggest that these changes may, at least in part, reflect particular properties of mutations to the first (5′) anticodon base, which is the site of nearly all (87%) isoacceptor shift anticodon mutations. Considering that groups of isoacceptor tRNAs can contain different antideterminants encoded by distinct sequence elements (Fender et al. 2004) and that the anticodon has a prominent place in most identity systems, we speculate that coordinate interactions between the 5′ anticodon base and the distinct identity elements of isoacceptors may drive the coevolution of both.

In recent years, a handful of studies have addressed the functional relevance of tRNA sequence variations in translation, independent of the tRNA's role in aminoacylation. Geslain and Pan (2010) showed that tRNAs with the same anticodon but different sequences (known as isodecoders), which are particularly common in mammalian genomes, have similar stability and aminoacylation activity but different stop-codon suppression activity and, thus, translational efficiency. By examining codon pair choices in open reading frames, Buchan et al. (2006) found evidence that nucleotide interactions between the tRNAs occupying ribosome A-site and P-site compartments influenced codon pair preference and translational performance, thus suggesting tRNA sequence elements, including, but not limited to, the anticodon, may be fine-tuned to alter ribosomal interactions. Saks and Conery (2007), studying the sequence conservation of bacterial tRNA genes grouped according to anticodon triplets, found that several bases were conserved in an anticodon-dependent manner rather than an identity-dependent manner. Such residues include the 32–38 pseudo-base pair at top of anticodon loop and residue 37, adjacent to the third anticodon nucleotide. In line with Buchan et al. (2006), the investigators suggest that structural interactions between the anticodon and other regions of the tRNA are important modulators of translational efficiency at the ribosome.

Since both aaRS interaction and ribosomal interaction constrain tRNA structure in a manner dependent on the anticodon, we expect mutations that coevolve with alloacceptor shift mutations to affect both identity elements and ribosomal interaction sites. In contrast, sites that covary with isoacceptor shifts should affect only the ribosomal interaction sites. Indeed, we find some agreement between the anticodon-dependent conservation data of Saks and Conery (2007) and the top covarying sites of the anticodon shifts, such as position 37 in the alloacceptor shifts and positions 32 and 44 in the isoacceptor shifts; most of the key sites of covariance among both categories are not those identified as conserved in an anticodon-dependent manner.

### Frequently covarying sites

While the top-scoring covarying mutation sites for both alloacceptor and isoacceptor shifts are spread out over the tRNA molecule, there are notable differences between the two classes. Among the most frequently covarying sites in alloacceptor shifts are the tip of the acceptor stem (bases 72 and 73) and the 3′ side of the anticodon loop (37, 37/38, and 39); these distal bases of the tRNA structure have been previously described as key identity determinants in a wide range of systems (Giege et al. 1998). In isoacceptor shifts, the same regions show very little covariance with anticodon substitutions. Thus, specific changes in the distal ends of the tRNA molecule are important and relatively common in the evolution of alloacceptor (but not isoacceptor) anticodon shifts. The only region that is strongly associated with covariation in both categories of anticodon shift is the variable loop; however, the precise location of mutations is different: base 45 in alloacceptor shifts and 44, 47/48, and 48 in isoacceptor shifts. The variable loop is important in determining tRNA-Ser (and thus selenocysteine) identity in both humans (Achsel and Gross 1993; Breitschopf and Gross 1994) and yeast (Himeno et al. 1997), yet there is only one anticodon shift involving a tRNA-Ser/SeC anticodon. The base pair of residues 27 and 43 is known to be involved in codon recognition (Yarus et al. 2003), and the base pair of residues 26 and 44 has been shown to conserved according to the anticodon (Saks and Conery 2007). Thus we speculate that the variable loop mutations may be involved in anticodon-dependent structural rearrangements to facilitate codon recognition, rather than aaRS interactions.

Interestingly, the sites in and around the anticodon loop that covary with alloacceptor anticodon mutations are all located on the 3′ side. When bound in the ribosome A-site, these nucleotides (37–39) face the closely adjacent P-site tRNA anticodon (*Thermus thermophilus* crystal structure: PDB accession nos. 1GIX and 1GIY) (Yusupov et al. 2001). Previous studies have found that chemical modifications of A-site tRNA nucleotides at these locations are important for reading frame maintenance and aminoacyl-tRNA selection (Li et al. 1997; Urbonavicius et al. 2001, 2003). In their analysis of codon pair biases, Buchan et al. (2006) suggested that nucleotide identity and chemical modifications of these nucleotides 3′ of the anticodon loop have a role in translation optimization, dependent on codon-pair usage. If the role of these nucleotides in translation optimization is related to the identities specified by codon pairs, it may explain why anticodon substitutions to the nucleotides 3′ of the anticodon loop are common during identity (alloacceptor) shifts, but not isoacceptor shifts.

### Many mutation pathways to identity change

A few covarying sites show preferences for certain synthetase classes and particular base substitutions. However, in general both alloacceptor and isoacceptor shifts show considerable heterogeneity in the extra-anticodon mutations that covary with their anticodon mutations. Many anticodon shifts involving the same transition of identities and anticodon seem to have very different mutations outside the anticodon. We also found at least two instances of extra-anticodon mutations that contradict known identity determinants. However, we note that for some eukaryotic tRNAs (Arg, Ile, and Lys), the acceptor stem is known not to contain major identity determinants (Senger et al. 1997; Delagoutte et al. 2000; Francin and Mirande 2006). In these tRNAs, as well as those for Asp, Leu, Met, Phe, and Ser, identity is thought to be controlled to greater or lesser extents by more complex structural features in other parts of the molecule (Sampson et al. 1989; Putz et al. 1991; Nazarenko et al. 1992; Senger et al. 1992, 1995; Achsel and Gross 1993; Breitschopf and Gross 1994; Breitschopf et al. 1995; Aphasizhev et al. 1997; Himeno et al. 1997). Furthermore, identity determinants for Asn and Gln tRNAs have not been identified in eukaryotes. Our knowledge of identity determinants is limited by the technical nature of experimental determination, which generally involves substituting particular residues, especially those in the acceptor stem, and swapping domains of different tRNAs. It is likely that identity rules vary in eukaryotes, as they are known to in bacteria (Freyhult et al. 2007). Thus, our results suggest that tRNA identity systems are governed by more complex and subtle rules than is currently appreciated. We base these conclusions on (1) the ubiquity and frequency of detected anticodon shifts, (2) the low average numbers of covarying mutations, (3) the conflicts with known eukaryotic identity rules reported, and (4) the dissimilar patterns of covarying mutations observed for the alloacceptor and isoacceptor shifts involving the same identities.

According to the “ambiguous identity” hypothesis proposed by Ardell and Andersson (2006), new identity rules may evolve through spontaneous mutations that create and pass through an intermediate state of ambiguous tRNA charging or translational efficiency. We speculate that the tRNA anticodon shifts we describe represent different stages of this process: Some genes we describe may be in an ambiguous state, while others have switched to exclusively charge the new anticodon-specified amino acid. We have also shown that alloacceptor anticodon shifts that involve a transition between aaRS classes are common. Class I and II aaRSs approach the tRNA molecule from different sides (Lapointe and Brakier-Gingras 2003). We can therefore extend the ambiguous identity hypothesis to include the possibility that both synthetases could bind to the same tRNA at the same time, facilitating the evolution of one state to the other. In general, the potential for a tRNA gene to undergo an identity switch must depend on a variety of interrelated factors: (1) both identity systems involved, including their determinants and anti-determinants; (2) the ribosome-interacting nucleotides of the tRNA, including the anticodon; and (3) the levels of genomic redundancy, including the numbers of isoacceptors in each family. Despite these complex and interdependent constraints, our findings suggest that the barriers to switching identity states are frequently surmounted.

## MATERIALS AND METHODS

### Mappings and ortholog set compilation

We used tRNAscan-SE (Lowe and Eddy 1997) in eukaryotic and bacterial mode, as appropriate, to annotate tRNAs in a total of 36 eukaryotic species from four different taxa: five primates, six nematode worms, 11 fungi, 12 *Drosophila* flies, and 61 bacterial species, each from a different genus of the Enterobacteriaceae family. The list of species and genome assembly versions is provided in Supplemental File 1, and we provide GFF lists of our tRNA annotations in Supplemental File 2. We used a flank-mapping method to detect regions of microsynteny conservation, as previously described (Rogers et al. 2010). Briefly, we extracted genomic flanking regions surrounding each tRNA. We masked all tRNAs in these genomic regions, together with all repeats using RepeatMasker (Smit et al. 1996–2010) and the 2011-09-20 edition repeat libraries (Jurka et al. 2005). We then used WU-BLASTN 2.0MP (Gish 1996–2004) to search the tRNA flanking regions against the tRNA flanking regions from all other genomes of that group, using the following parameters: word size, 7; E-value threshold, 10^−6^; and the hspsepSmax and hspsepQmax parameters, 50 bases. A range of flanking sequence lengths were tested for each taxonomic group; the length used for the analysis was the length at which an increased size greatly increased the number of mappings. These lengths were 5 kb upstream and downstream in primates, nematodes, and flies and 1 kb upstream and downstream in Saccharomycetes and Enterobacteriaceae. The tRNAs in each taxonomic group with flanking sequence BLAST hits with E-values <10^−6^ were assembled into sets of orthologs by single linkage clustering. Since the aim is to detect anticodon shifts rather than to analyze rates of tRNA duplication and deletion, unlike in our previous work (Rogers et al. 2010), we use a “relaxed” definition of the ortholog set concept, which potentially includes closely related in- and out-paralogs.

### Anticodon shift detection and validation

We selected all ortholog sets containing more than one anticodon as potential anticodon shifts. We used the following criteria to further conservatively filter the data set:

1. We discarded anticodon shifts involving tRNAs annotated by tRNAscan-SE with a score of <50 bits. This threshold was determined by manual inspection of alignments of the low-scoring tRNAs involved in anticodon shifts: scores <50 bits were often characterized by structures with large truncations or insertions in usually nonvariable regions and by several substitutions that do not conserve base-pairings in stem regions. Thirty-four potential anticodon shifts were discarded at this step, 22 of them from primates.
2. Anticodon shifts involving sequence pairs that are mutually widespread across species’ tRNA complements are difficult to prove authentic, rather than the result of spurious mappings. We therefore discarded potential shifts that were inferred by Dollo parsimony (Farris 1977) to have arisen before divergence of the taxon group, based on the presence and absence of tRNAs of both anticodons in the various species, and topology of the genus tree. In cases where lack of mappings made this test uninformative, we searched a representative gene of the shifting tRNA type against the Transfer RNA Database (Juhling et al. 2009). If the shifting genes resulted in identical or nearly identical matches outside the taxon group, the anticodon shift was discarded.
3. We aligned the tRNA sequences of each ortholog set using ClustalW 2.0.12 (Thompson et al. 1994) and viewed the alignment in Jalview (Clamp et al. 2004). We counted the number of mutations between the sequences of tRNAs with the potential anticodon shift. Sequence pairs with more than 10 mutations were discarded as potential false ortholog calls.
4. For potential alloacceptor shifts only, the following criterion was used to override discard according to tests 2 and 3. We searched a representative gene of each identity against Tfam 1.3 (Taquist et al. 2007), which classifies tRNA genes based on sequence motifs outside the anticodon using known identity rules. If the shifting identity tRNA is predicted to have the same identity as its putative ortholog, rather than the identity specified by its own anticodon, this is taken as a confirmation of the shift and it is retained.

### Coevolution analysis

In order to find which sites were coevolving with the mutations in the anticodon, we generated alignments of tRNAs for each validated anticodon shift by aligning the sequences against tRNAscan-SE's eukaryotic covariance model (Lowe and Eddy 1997) using INFERNAL 1.0.2 (Nawrocki et al. 2009). Alignment columns were numbered according to the Sprinzl et al. (1991) consensus tRNA numbering. Insertions with respect to the covariation model consensus were numbered x.01, x.02, etc.

For each alignment, we assessed the coevolution of the anticodon with other sites in the tRNA sequence using the quantity MI (Chiu and Kolodziejczak 1991; Gutell et al. 1992). The MI between two columns reflects the degree to which the pattern in the two columns is correlated. If bases occur independently at the two sites, the theoretical value for MI is zero. We used standard MI normalized by joint entropy of the two positions *H*(*X|Y*). The effect of this normalization is to make full dependency between the two sites score 1, regardless of the relative numbers of sequences of each tRNA type present. Normalization by pair entropy has been shown to perform best out of several variations of MI in detection of coevolving positions in protein sequences (Martin et al. 2005).

For each extra-anticodon alignment position with a site entropy *H*(X) greater than zero, we calculated the MI with respect to each of the three anticodon positions; the highest score of the three was taken as the site's MI with respect to the anticodon. The method treats consensus positions and nonconsensus positions uniformly, so substitutions, insertions, and deletions are all potentially detectable as covarying mutations. We summed the scores at each position for all alloacceptor shifts and isoacceptor group shifts, as well as alloacceptor shifts within both synthetase classes and transitions between classes I and II. Structure “heat maps” for each class of shift were drawn using VARNA (Darty et al. 2009).

## SUPPLEMENTAL MATERIAL

Supplemental material is available for this article.
